# Development and validation of a diagnostic nomogram model for predicting monoclonal gammopathy of renal significance

**DOI:** 10.1038/s41598-023-51041-z

**Published:** 2024-01-10

**Authors:** Yijun Dong, Ge Yan, Yiding Zhang, Yukun Zhou, LiYang Zhu, Jin Shang

**Affiliations:** 1 Department of Nephrology, The First Affiliated Hospital of Zhengzhou University, Zhengzhou, Henan China; 2 School of Medicine, Zhengzhou University, Zhengzhou, Henan China; 3 Laboratory Animal Platform of Academy of Medical Sciences, Zhengzhou University, Zhengzhou, Henan China

**Keywords:** Diseases, Medical research, Nephrology, Risk factors

## Abstract

In patients with kidney disease, the presence of monoclonal gammopathy necessitates the exploration of potential causal relationships. Therefore, in this study, we aimed to address this concern by developing a nomogram model for the early diagnosis of monoclonal gammopathy of renal significance (MGRS). Univariate and multivariate logistic regression analyses were employed to identify risk factors for MGRS. Verification and evaluation of the nomogram model's differentiation, calibration, and clinical value were conducted using the receiver operating characteristic (ROC) curve, calibration curve, and decision curve analysis. The study encompassed 347 patients who underwent kidney biopsy, among whom 116 patients (33.4%) were diagnosed with MGRS and 231 (66.6%) with monoclonal gammopathy of undetermined significance. Monoclonal Ig-related amyloidosis (n = 86) and membranous nephropathy (n = 86) was the most common renal pathological type in each group. Notably, older age, abnormal serum-free light chain ratio, and the absence of microscopic hematuria were identified as independent prognostic factors for MGRS. The areas under the ROC curves for the training and verification sets were 0.848 and 0.880, respectively. In conclusion, the nomogram model demonstrated high accuracy and clinical applicability for diagnosing MGRS, potentially serving as a valuable tool for noninvasive early MGRS diagnosis.

## Introduction

Monoclonal immunoglobulinemia (MIg) constitutes a highly heterogeneous group of diseases, ranging from monoclonal gammopathy of undetermined significance (MGUS) to clearly diagnosed lymphoplasmacytic lymphomas. This spectrum represents a dynamic pathophysiological process. The concept of monoclonal gammopathy of renal significance (MGRS) was introduced in 2012^1^. While hematological changes in MGRS lesions resemble those in MGUS lesions, the presence of MIg in MGRS lesions can lead to kidney damage. Unlike MGUS lesions, initiating treatment for most MGRS cases upon diagnosis is crucial to prevent further kidney damage^2^. Therefore, determining whether renal injury is associated with MIg holds considerable importance for treatment decisions and patient prognosis assessments. Although various prediction models have been employed for clinical prediction and prognostic assessment in various diseases^3,4^, prediction models for the diagnosis of MGRS lesions are notably absent in existing literature. There, in this study, we aimed to conduct a retrospective investigation into the correlation between clinical indicators and the diagnosis of MGRS lesions. Furthermore, we developed a non-invasive diagnostic model for MGRS lesions using a nomogram. Our findings are anticipated to enhance clinical understanding by providing a valuable diagnostic tool for MGRS lesions, enabling timely interventions to mitigate kidney damage and improving patient outcomes.

## Results

### Baseline characteristics and pathologic manifestations of patients (MGRS versus MGUS)

Types and distribution of monoclonal immunoglobulins in studied groups are provided in Supplemental Table S1. In the MGRS group, MIg-related amyloidosis was the most common renal pathological type, constituting over half of all MGRS lesions (n = 86, 74%). Cast nephropathy was the second most common kidney lesion (n = 14; 12%), followed by light chain deposition disease (LCDD) (n = 9; 7.8%). Immunopathological examination revealed that light chain λ deposition was the main type of light chain renal amyloidosis. Conversely, the most common type in the MGUS group was membranous nephropathy (n = 86, 37.2%), followed by immunoglobulin A nephropathy (n = 23, 9.9%), diabetic nephropathy (n = 20, 8.6%), and tubulointerstitial nephropathy (n = 19, 8.2%). Notably, several glomerular diseases, such as IgAN, MN and MPGN may be associated with MIgs. But no deposition of MIg was observed in the kidneys of our MGUS group using immunofluorescence. In addition, we found 72 patients with MN and 2 patients with atypical MN displayed positive serum PLA2R staining. Patients with atypical MN are usually characterized by positive C1q deposition, mesangial and endothelial cell proliferation, and deposits of subendothelial and subepithelial electron dense materials of pathological characteristics; however, no definite underlying causes of MN could be identified. Further details regarding other pathological types are provided in Supplemental Table S2.

Comparison between MGUS and MGRS lesions revealed distinctive differences (Table 1). Patients with MGRS were significantly less likely to have hypertension, demonstrated a lower incidence of microscopic hematuria and lower serum c-reactive protein levels. Moreover, patients with MGRS lesions were older, presented with higher 24-h urinary protein levels, elevated triglyceride levels, and were more likely to exhibit nausea. We also found that patients in the MGRS group had a significantly higher abnormal free light chain (FLC) ratio. Additional details are provided in Table 1.

**Table 1:** Baseline characteristics of patients with MG who underwent kidney biopsy.

Clinical indices	MGUS Lesions (n = 231)	MGRS Lesions (n = 116)	*p* value
Male, n (%)	78 (33.8)	39 (33.6)	1
Age, years	55.13 (12.73)	60.18 (9.22)	< 0.001
Edema, n (%)	180 (77.9)	86 (74.1)	0.537
Nausea, n (%)	46 (19.9)	40 (34.5)	0.01
Hypertension, n (%)	126 (54.5)	47 (40.5)	0.034
Hematuria	113 (48.9)	30 (25.9)	< 0.001
Hemoglobin (g/L)	117.20 (22.76)	118.18 (25.67)	0.717
BUN (mmol/L)	12.39 (21.89)	9.37 (7.18)	0.15
Creatinine (μmol/L)	152.32 (142.43)	150.49 (164.71)	0.915
UA (μmol/L)	365.95 (123.72)	365.60 (196.61)	0.984
Albumin (g/L)	29.57 (8.72)	27.81 (8.67)	0.076
eGFR (mL/min per 1.73 m2)	68.66 (72.89)	67.84 (84.29)	0.161
24hTP (g/day)	4.60 (4.15)	5.52 (3.62)	0.043
CRP (mmol/L)	12.18 (29.80)	5.98 (10.71)	0.035
ESR (mm/h)	46.55 (36.70)	50.61 (35.49)	0.343
Complement 3 (g/L)	1.11 (0.29)	1.16 (0.31)	0.173
Complement 4 (g/L)	0.28 (0.12)	0.31 (0.14)	0.087
Abnormal FLC ratio	38 (16.5)	87 (75.0)	< 0.001
dFLC (mg/L)	62.13 (320.37)	874.79 (2220.76)	< 0.001

Values of continuous variables are described as mean ± SD or median (interquartile range) depending on the distribution, and categorical variables described as count (%). Hematuria positive urinary red blood cells > 5.

*BUN* Serum urea nitrogen, *UA* serum uric acid, *eGFR* estimated glomerular filtration rate, *24hTP* 24-h urine total protein, *CRP* C-reactive protein, *ESR* erythrocyte sedimentation rate, *FLC* free light chain, *dFLC* free light chain difference.

### Baseline characteristics of patients in training group and validation group

The laboratory and clinical data of the training and validation groups are presented in Table 2. In total, 347 patients were enrolled in the study. Among them, 244 patients were randomly assigned to the training group, whereas the remainder formed the validation set. The mean age of patients in the training set was 56.73 ± 11.70 years, with 75.7% being male. In the validation set, the patients’ mean age was 57.04 ± 12.42 years, and males accounted for 79.6%. Notably, the only significant difference between the training and validation sets was observed in total cholesterol levels (P < 0.05). Other clinical indicators did not exhibit significant differences between the two groups (P > 0.05), as shown in Table 2.

**Table 2:** Baseline characteristics of patients in Validation group and Training group.

Clinical indices	Validation group (n = 103)	Training group (n = 244)	*p* value
Age, years	57.04 (12.42)	56.73 (11.70)	0.825
Edema, n (%)	82 (79.6)	184 (75.7)	0.519
Nausea, n (%)	28 (27.2)	58 (23.9)	0.605
Hypertension, n (%)	48 (46.6)	125 (51.4)	0.481
Hematuria	40 (38.8)	103 (42.2)	0.642
Hemoglobin (g/L)	118.97 (25.28)	116.92 (23.10)	0.466
BUN (mmol/L)	9.31 (5.33)	12.26 (21.64)	0.173
Creatinine (μmol/L)	140.39 (118.83)	156.51 (161.34)	0.362
UA (μmol/L)	369.54 (202.62)	364.27 (124.66)	0.768
Albumin (g/L)	27.87 (8.40)	29.45 (8.84)	0.124
eGFR (mL/min per 1.73 m^2^)	65.18 (33.66)	67.98 (71.48)	0.704
24hTP (g/day)	4.91 (3.46)	4.91 (4.21)	1
TC (mmol/L)	6.20 (3.07)	5.56 (2.32)	0.039
CRP (mmol/L)	5 (4.9)	13 (5.3)	1
ESR (mm/h)	9 (8.7)	14 (5.7)	0.429
Complement 3 (g/L)	1.23 (0.27)	1.26 (0.29)	0.185
Complement 4 (g/L)	0.26 (0.11)	0.28 (0.13)	0.079
Abnormal FLC ratio	42 (40.8)	83 (34.0)	0.282
dFLC (mg/L)	10 (9.7)	30 (12.3)	0.613

*TC* total cholesterol.

### Establishment of the diagnostic nomogram model

**Figure 1:**
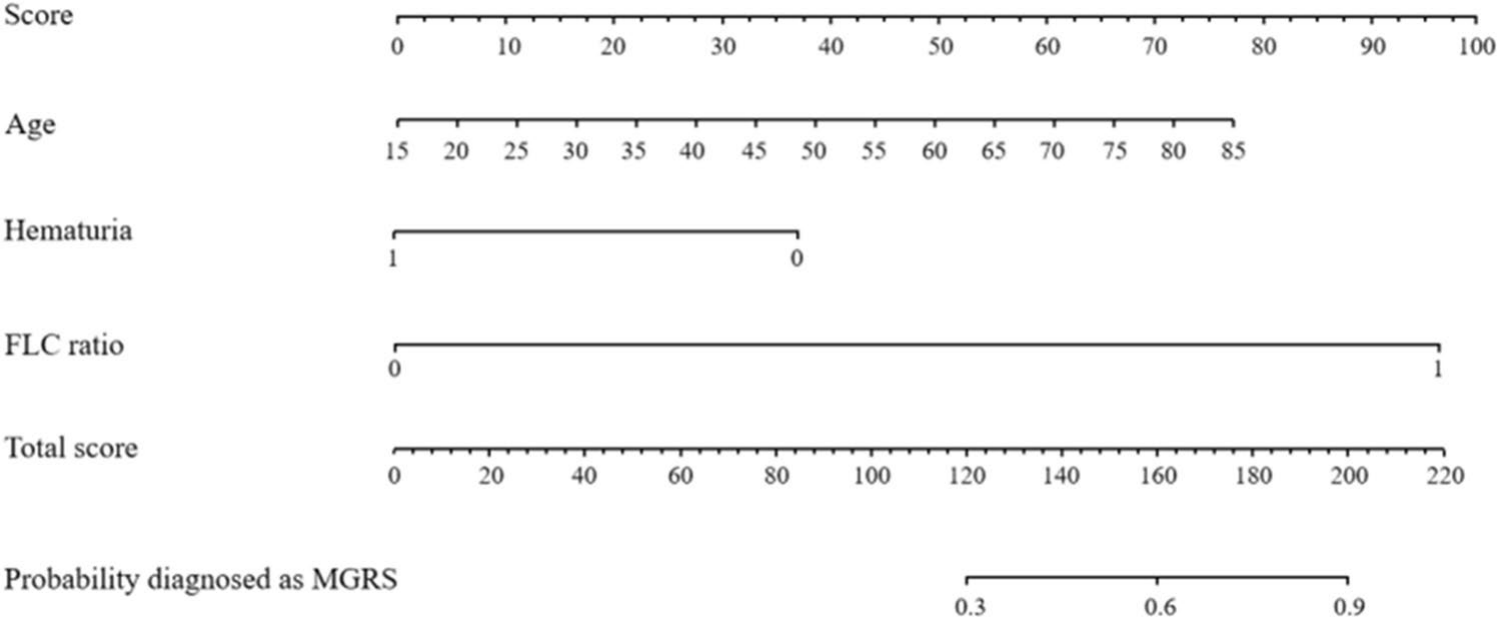
Nomogram predicting MGRS consisting of abnormal FLC ratio, older age and the absence of microscopic hematuria.

Univariate analysis revealed that clinical indicators, including age, edema, and nausea, were significantly associated with MGRS lesions. Collinearity among all factors were analyzed, and after excluding collinearity, the aforementioned factors were subjected to a multivariate logistic regression analysis. However, the results indicated that only age [odds ratio (OR) 1.048, 95% Confidence Interval (CI) 1.008–1.089, P = 0.018], abnormal serum-free light chain ratio (OR 0.093, 95%CI 0.04–0.218, P < 0.001), and absence of hematuria (OR 3.275, 95% CI 1.396–7.681, P = 0.006) were independent prognostic factors for MGRS lesions. Details are presented in Table 3. The scores for the independent variables were assigned according to the regression coefficients and are displayed as nomograms in Fig. 1.

**Table 3:** Potential risk factors identified by univariate and multivariate logistic regression analysis in training group.

Clinical indices	Univariable			Multivariable		
	OR	CI	*p* value	OR	CI	*p* value
Male, yes	1.251	0.711–2.201	0.437			
Age, 1 year	1.036	1.009–1.063	0.008	1.048	1.008–1.089	0.018
Edema, yes	1.551	0.835–2.878	0.165			
Without nausea, yes	0.38	0.206–0.702	0.002	0.417	0.172–1.012	0.053
Hypertension, yes	2.521	1.429–4.447	0.001	1.507	0.669–3.395	0.323
Without hematuria, yes	2.086	1.164–3.738	0.013	3.275	1.396–7.681	0.006
Hemoglobin, 1 g/L	0.996	0.985–1.008	0.541			
BUN, 1 mmol/L	0.983	0.954–1.013	0.269			
Creatinine, 1 μmol/L	1.001	0.999–1.002	0.398			
UA, 1 μmol/L	0.999	0.997–1.001	0.397			
Albumin, 1 g/L	0.98	0.949–1.011	0.201			
eGFR, 1 mL/min per 1.73 m2	0.998	0.992–1.004	0.508			
24hTP, 1 g/d	1.047	0.982–1.115	0.16			
CRP, 1 mmol/L	0.984	0.963–1.006	0.161			
ESR, 1 mm/h	1.005	0.997–1.012	0.226			
Complement 3, 1 g/L	2.004	0.764–5.254	0.158			
Complement 4, 1 g/L	7.811	0.836–72.984	0.071	2.833	0.108–74.355	0.532
Normal FLC ratio, yes	0.064	0.033–0.125	< 0.001	0.093	0.04–0.218	< 0.001
dFLC, 1 mg/L	1.002	1–1.003	0.008	1.001	1–1.001	0.094

Hematuria positive urinary red blood cells > 5.

*BUN* serum urea nitrogen, *UA* serum uric acid, *eGFR* estimated glomerular filtration rate, *24hTP* 24-h urine total protein, *CRP* C-reactive protein, *ESR* erythrocyte sedimentation rate, *FLC* free light chain, *dFLC* free light chain difference.

Based on hematologic indexes. Hematuria: 0 without hematuria, 1 hematuria; FLC ratio: 0 normal FLC ratio, 1 abnormal FLC ratio. The usage of the nomogram is illustrated in an 70-year-old male patient with abnormal FLC ratio and hematuria. According to the nomogram, points for age, hematuria and abnormal FLC ratio were 62.5, 0 and 96.25, respectively. The total point added up to 158.75 for this patient, which represented approximately 0.60 of MGRS probability (indicated in the nomogram).

### Differentiation, calibration and clinical applicability of the diagnostic nomogram model

**Figure 2:**
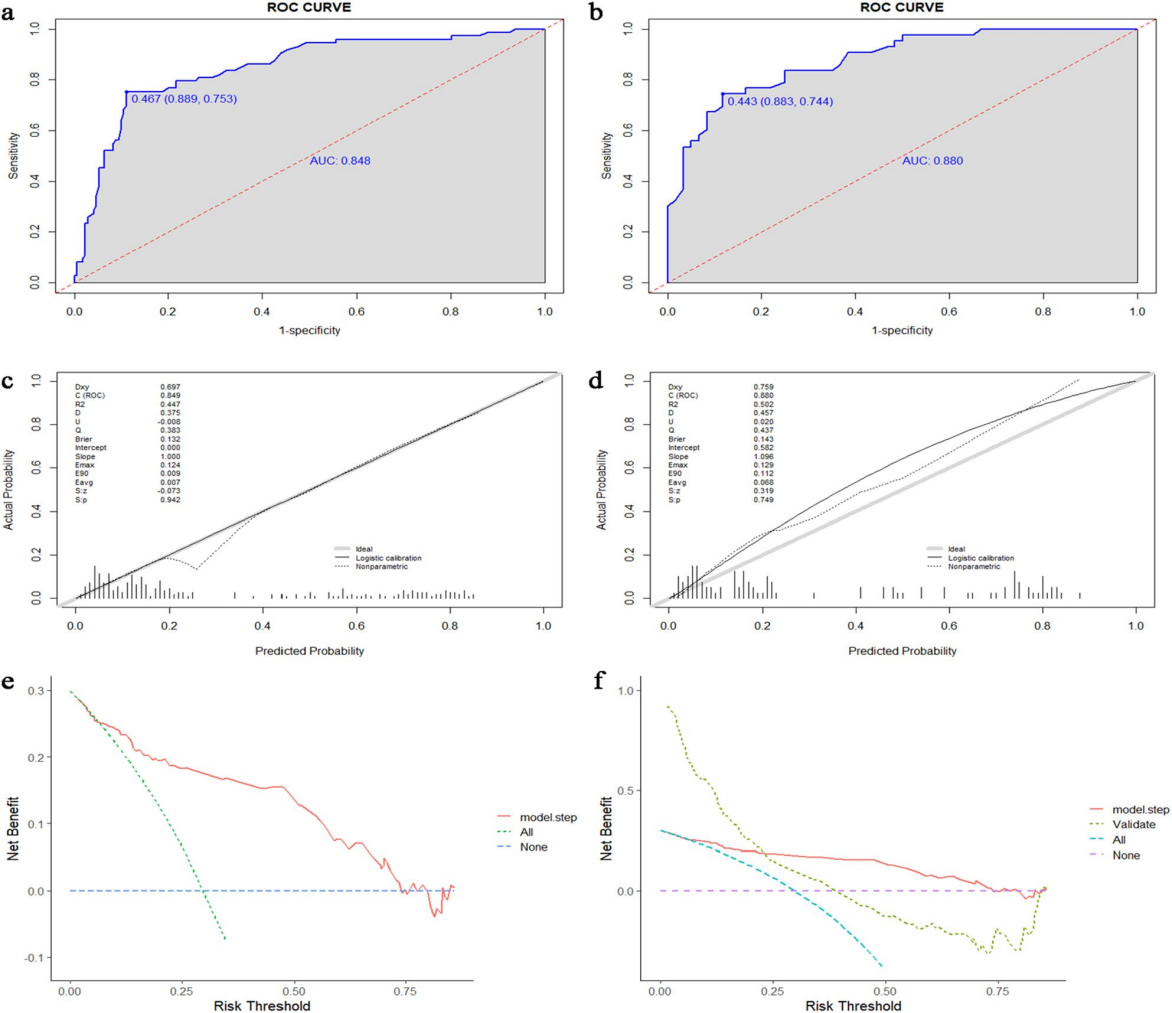
The area under the ROC curve (AUC) demonstrate the prognostic accuracy in predicting MGRS lesions both in training cohort (**a**) and validation cohort (**b**). Calibration curves depict the calibration of each model in terms of the agreement between the predicted probabilities and observed outcomes in the training cohort (**c**) and validation cohort (**d**). Decision curve analysis demonstrate the clinical utility in predicting MGRS lesions in the training cohort (**e**) and validation cohort (**f**).

To validate the efficacy of our nomogram model, we employed ROC analysis. In the training set, the area under the curve (AUC) was 0.848 (95% CI 0.7936–0.9034) using the bootstrap internal verification method (n = 1000) (Fig. 2a). Notably, the sensitivity and specificity of the training set were 88.9% and 75.3%, respectively. In the validation set, the model displayed an AUC of 0.880 (95% CI 0.815–0.9443), with sensitivity and specificity of 88.3% and 74.4%, respectively (Fig. 2b). These AUC values highlight the strong diagnostic predictive performance of the nomogram.

Furthermore, the calibration curve illustrated the high accuracy of the nomogram model in both the training (Fig. 2c) and validation cohorts (Fig. 2d). The 45-degree line represent the standard line, with the broken lines closely aligned, indicating a homogeneous distribution of predicted points (P > 0.05). A decision curve analysis (DCA) was performed to evaluate the prediction models by calculating the net clinical benefit. In the DCA curves, the x-axis represents the threshold probability, whereas the y-axis represents the net benefit, obtained by subtracting the disadvantage from the advantage. The DCA curves (Fig. 2e,f) reveal that the nomogram demonstrated enhanced diagnostic efficacy for MGRS lesions in both the training and validation cohorts, indicating its clinical effectiveness.

## Discussion

The concept of MGUS was first proposed by Robert Kyle in 1978, and it was defined as a "precancerous state.” The incidence of MGUS varies greatly based on age and ethnicity, with lower rates in Asia (approximately 0.4% to 2%)^5,6^ than in North America and Northern Europe. A study reported an MGUS incidence rate of 6.1% in the United States^7^. The prevalence of MGUS tends to increase with age, reaching approximately 1.7% in people aged 50–60 years and 6.6% in those older than 80 years^8^. In 2012, the International Kidney and Monoclonal Gammopathy Research Group (IKMG) introduced the concept of the MGRS. However, the incidence and prevalence of MGRS remain unclear. In this study, we conducted a comprehensive retrospective MGRS cohort study involving 347 patients with MIg who underwent kidney biopsy. Among them, 116 (33.4%) exhibited MGRS lesions, consistent with results of previous reports^8,9^. According to previous studies, MGRS constitutes 2–10% of cases involving monoclonal gammopathy and kidney injury^10–12^. Kidney damage and MIg may either coexist or be causally related. A study conducted by Paueksakon^13^ revealed that 45% of patients initially suspected of having MGRS lesions had no MGRS-related lesions. Renal needle biopsy remains the gold standard for diagnosing MGRS. However, its invasiveness and the need for high clinical accuracy limit its widespread clinical application, with only approximately 2.5% of patients with MIg undergoing kidney biopsy^8^. Therefore, exploring a noninvasive diagnostic model for MGRS is of great clinical significance.

In recent years, nomogram prediction models have been developed to predict the probability of an outcome event by comprehensively analyzing multiple related factors. These models have significantly contributed to early diagnosis and prognostic assessments across various diseases^14,15^. However, no diagnostic nomogram model has been developed for MGRS. Initially, we identified abnormal FLC ratio (κ/λ), age, and absence of hematuria as indicators for MGRS risk through logistic regression analysis. Although similar, these findings diverged slightly from previous findings^8,9^. Subsequently, we integrated these three factors into a nomogram, visually representing them on a single plane with calibrated line segments, thereby enabling the calculation of a total score based on their values. Higher scores correlated with a greater likelihood of an MGRS diagnosis. Among the hematologic indices, an abnormal FLC ratio was the most robust predictor for detecting MGRS lesions in the multivariate analysis, followed by age, consistent with previous research findings^9^. Unlike the University of Minnesota study^8^, our findings did not show a correlation between hematuria and an increased likelihood of MGRS. Another study by a Chinese scholar observed a lower percentage hematuria in the MGRS group^9^. One plausible explanation for this discrepancy is that, compared with Western countries, Chinese patients exhibit a higher prevalence of amyloidosis^16^, accounting for nearly 76% of the cases in our MGRS group. Amyloidosis predominantly manifests as nephrotic syndrome with infrequent hematuria^17^, suggesting that hematuria may not be a reliable predictive indicator of MGRS in Chinese patients. Notably, an abnormal FLC ratio mostly indicates amyloid light-chain (AL)-amyloidosis, often presenting as a light-chain MGUS. Therefore, this predictive tool may predominantly apply to AL-amyloidosis, which is prevalent in most MGRS cases. Ideally, distinct models predicting AL-amyloidosis/MGRS (termed amyloid MGRS) and non-amyloid MGRS glomerulopathies would be preferable. However, owing to the limited number of non-amyloid MGRS cases, constructing such separate models remains challenging.

Patients in MGRS group were older and showed more urinary protein in Table 1. Consistent with previous research findings^9,18^, amyloid nephropathy was the most common type of MGRS lesion in our cohort, followed by cast nephropathy. Of the 13 cases of thrombotic microangiopathy (TMA), 1 case was identified as MIg-associated TMA following the exclusion of other causes, whereas the remaining 12 cases were attributed to malignant hypertension. MIg-associated TMA and C3 glomerulopathy are special MGRS lesions, which are associated with indirectly inferring the complement system by MIg instead of renal deposition of MIg^19^. Patients with TMA and C3 glomerulopathy combined with MIg can be diagnosed as MGRS after exclusion of other causes, such as diarrhea positive hemolytic uremic syndrome, malignant hypertension, or thrombotic thrombocytopenia purpura. A retrospective study revealed that 21% of patients with TMA aged over 50 years exhibited associated MIg^20^. In the MGUS lesion group, membranous nephropathy (MN) was the most common histopathological type, as opposed to arteriosclerosis, according to the Mayo Clinic study. This divergence may be due to the prevalence of both MN and monoclonal gammopathy in older individuals, with MN being more common in China^21^. IgAN accounted for 9.95% of MGUS, where 5 of which had serum monoclonal IgA. Among the MGUS cases, 14 (6.06%) involved MPGN. A previous study revealed that proliferative glomerulonephritis with monoclonal IgG deposits (PGNMID), first discovered by Nasr in 2004 and also known as Nasr’s kidney disease^22^, constituted 0.17% of cases in patients who underwent kidney biopsy^23^. However, PGNMID was not observed in our study, likely owing to its low incidence and the challenges associated with detecting MIg light chains through immunofluorescence in conventional frozen sections^24^. Moreover, there are no reported associations between MN and MIgs in our study. Of the 86 patients with MN testing negative for κ and λ light chains, 72 displayed positive PLA2R staining. Additionally, we observed 12 cases of atypical MN, 2 of which exhibited positive serum PLA2R staining. Huang^25^ highlighted differences in the antibody response between the early and later stages of MN, with IgG1 dominant in the early-stage deposits, eventually replaced by IgG4 as the dominant immunoglobulin in the later stages. Another study reported initial renal biopsies showing IgG polyclonal deposits, followed by subsequent biopsies showing IgG3 lambda monoclonal deposits^26^. However, in our case, renal biopsy was only performed during the early disease stage, with no subsequent biopsies. Consequently, it remains unclear whether the patient's IgG subtype converted to monoclonal or remained polyclonal. Therefore, long-term follow-up and monitoring are required for certain patients exhibiting a high clinical suspicion of MGRS in our study, particularly those with MIg-unrelated MN and MIg-unrelated MPGN.

Our study had several limitations. First, this was a retrospective study, and inherent selection bias could not be addressed. Second, perhaps challenges in detecting MIg in MN, IgAN, MPGN and so on, non-proliferative types of MGRS are in the majority in our study. The developed prediction tool may primarily be suitable for cases of AL-amyloidosis, which are prevalent in most MGRS cases. Finally, being a single-center study, all participants were recruited from the First Affiliated Hospital of Zhengzhou University. Consequently, the findings may not be representative of the entire Chinese population and might have a limited scope of applicability.

In summary, MGRS is a common and significant contributor to kidney injury in patients with monoclonal gammopathy. The diagnostic nomogram model developed in this study proves effective in predicting MGRS lesions. This non-invasive prediction tool also aids in identifying high-risk patients who require more proactive therapeutic interventions and aggressive follow-up schemes.

## Materials and methods

### Study design and population

**Figure 3:**
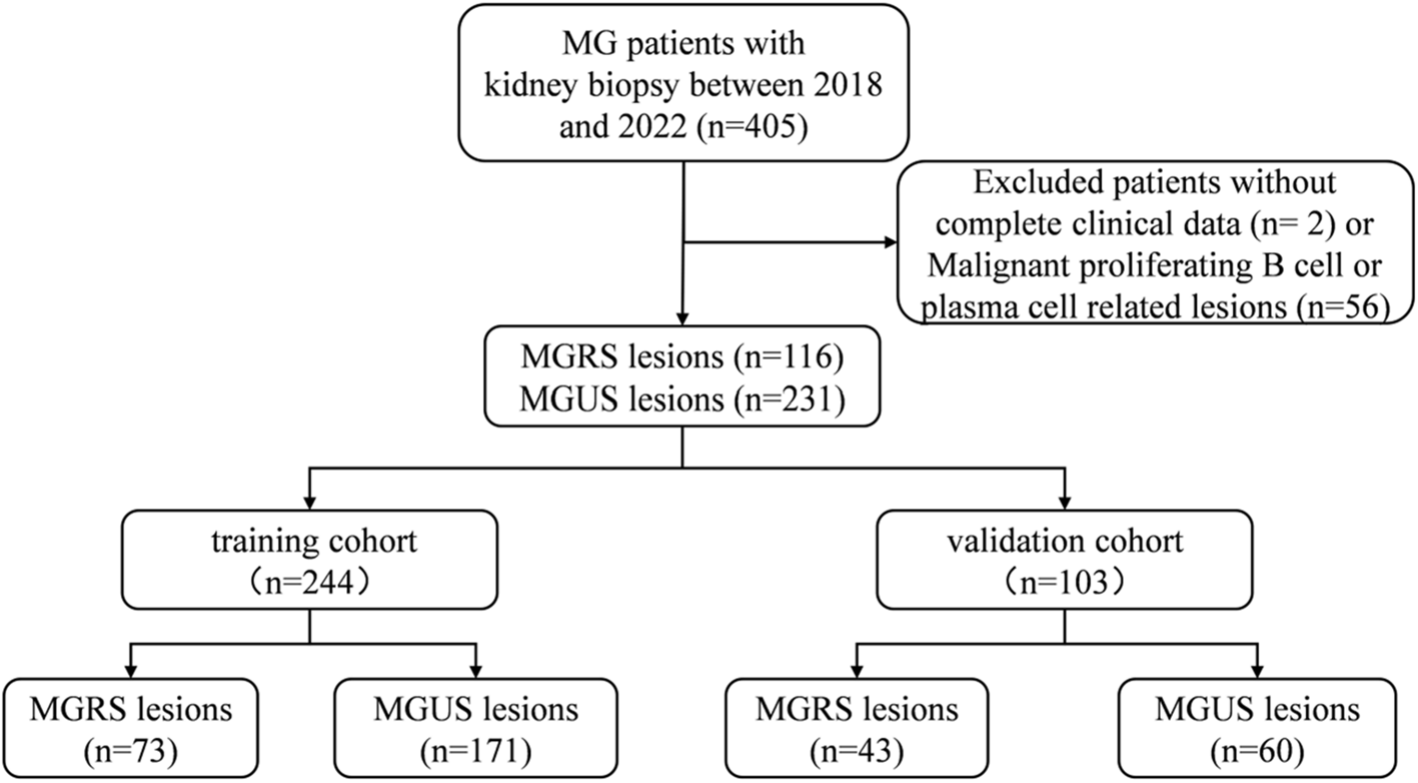
Study flow chart of patients with monoclonal gammopathy (MG) who underwent kidney biopsies from 2018 to 2022. MGRS, monoclonal gammopathy of renal significance; MGUS, monoclonal gammopathy of undetermined significance.

This is a retrospective study. Firstly, we identified all patients that had at least one positive serum or urine monoclonal results tested in the First Affiliated Hospital of Zhengzhou University from January 1, 2018 to October 31, 2022. Patients were considered to have monoclonal gammopathy (MG) if they have positive following tests, including serum monoclonal Ig electrophoresis, serum immunofixation, and blood FLC performed by immunoturbidimetry (sheep anti-human polyclonal antibody kit, Sebia, France), with or without positive urine protein electrophoresis or urine immunofixation test. We picked out those who underwent kidney biopsy in the First Affiliated Hospital of Zhengzhou University. Patients who met hematologic criteria of multiple myeloma, Waldenström macroglobulinemia, chronic lymphocytic leukemia, or other hematologic malignancies were excluded, before or at the time of kidney biopsy (Fig. 3).

The diagnosis of MGRS was based on the 2012 consensus proposed by the International Kidney and Monoclonal Gammopathy Research Group. MGUS were classified in line with the diagnostic criteria of MGRS, except no evidence of MG deposition was found in renal pathological examination. A total of 347 patients were enrolled. Based on the R-language caret package, all selected patients were randomly divided into the training set and the validation set at 7:3 ratio. A total of 244 patients were included in the training set, including 171 MGUS and 73 MGRS, and 103 patients in the validation set, including 60 MGUS and 43 MGRS. The details for patient selection are shown in Fig. 3. We confirm that informed consent was obtained from all participants and/or their legal guardians. This study was approved by the Ethics Committee of the First Affiliated Hospital of Zhengzhou University (approval number: 2022-KY-1493-001) and in compliance with the Declaration of Helsinki.

### Clinical/laboratory and kidney histopathology assessment

We performed comprehensive review of the baseline clinical and laboratory data at kidney biopsy. When eGFR ≥ 60 mL/min/1.73 m^2^, the ratio outside the range of 0.27–1.65 was considered as abnormal free light chain ratio. But when eGFR < 60 mL/min/1.73 m^2^ the ratio outside the range of 0.37–3.10 was considered abnormal^27^. The eGFR was obtained by the Chronic Kidney Disease Epidemiology Collaboration equation. Hematuria was defined if there were more than five red blood cells per high power field. Proteinuria was defined as having an 24-h urinary protein of > 300 mg.

Renal pathological examination included light microscopy, electron microscopy and immunofluorescence. The deposition of IgG, IgM, IgA, C3, C1q, light chain κ and λ in renal tissue was detected by direct immunofluorescence method. Results were observed under fluorescence microscope. Kidney biopsy specimens for light microscopy were fixed in 4% buffered formaldehyde. Histologic staining included hematoxylin and eosin, periodic acid–silver methenamine, periodic acid–Schiff staining, Masson trichrome and Congo red staining. Kidney tissue was fixed with cold glutaraldehyde (3.75%) for electron microscope observation. After sample preparation, the copper mesh was observed and photographed under Japanese Electron JEOL1400 plus transmission electron microscopy. As for detection and differentiation of MIg, SPE was performed by capillary electrophoresis, IFE was visualized by agarose gel electrophoresis, and blood FLC was performed by immunoturbidimetry, according to the instructions of the sheep anti-human polyclonal antibody kit (Sebia, France).

### Establishment and verification of nomogram model

First, univariate Logistic regression equation was used to analyze whether age, edema, hematuria, abnormal free light chain κ/λ ratio, dFlc and other indicators were influential factors in the diagnosis of MGUS, and collinear diagnosis was performed on all independent variables^28^. After excluding the collinearity problem, the independent variables with P < 0.1 were incorporated into the multi-factor Logistic regression equation by step-up analysis method. In order to facilitate clinical application, we used Stata 15, R3.5.1 to assign values to each risk factor (abnormal FLC ratio, age and absence of hematuria) and draw corresponding rows (Fig. 1), which is similar to a simple medical calculator. The specific method is as follows: (1) The value of each variable in the figure corresponds to a corresponding score in the first row; (2) Add the corresponding scores of all variables to get the total value; (3) Project the obtained total value to the last line to obtain the corresponding probability value. In the training set, the model was internally verified by Bootstrap (n = 1000), which provided stable estimates with low bias^29^. The area under receiver Operating characteristic curve (ROC) curve (AUC), calibration curve and decision curve analysis (DCA) were used to verify and evaluate the differentiation, calibration and clinical practicability of the model.

### Statistical analyses

R 3.5.1 software was used for statistical processing of the data. The measurement data of normal distribution were shown by x ± s, and the independent sample t test was used to compare the two groups. Measurement data of non-normal distribution represented by M (1/4, 3/4) and comparison between the two groups was conducted by Mann⁃ Whitney U test; Qualitative data were expressed as number of cases (percentage) and compared between the two groups using the Chi-square test or Fisher exact probability method. P < 0.05 was considered statistically significant.

### Supplementary Information

Supplementary Tables.

## Data Availability

The datasets used and analysed during the current study available from the corresponding author on reasonable request.
