# Supplementary material for: Development and validation of a diagnostic nomogram model for predicting monoclonal gammopathy of renal significance
Source: Sci Rep. 2024 Jan 10;14:990. doi: 10.1038/s41598-023-51041-z (PMC10781706; doi:10.1038/s41598-023-51041-z)
Supplement: Supplementary file 1 — Supplementary Tables. [file 41598_2023_51041_MOESM1_ESM.doc]

Supplemental Table 1. Types and distribution of monoclonal immunoglobulins in studied groups

|  | MGRS Lesions(n=116) | MGUS Lesions(n=231) |
| --- | --- | --- |
| IgA-κ | 1 | 9 |
| IgA-λ | 3 | 11 |
| IgM-κ | 2 | 6 |
| IgM-λ | 1 | 4 |
| IgG-κ | 6 | 15 |
| IgG-λ | 7 | 13 |
| IgD-κ | 1 | 0 |
| IgD-λ | 1 | 0 |
| κ | 24 | 2 |
| λ | 48 | 5 |
| IgA-κ accompanied with κ | 2 | 25 |
| IgA-λ accompanied with λ | 3 | 29 |
| IgM-κ accompanied with κ | 1 | 13 |
| IgM-λ accompanied with λ | 0 | 7 |
| IgG-κ accompanied with κ | 7 | 50 |
| IgG-λ accompanied with λ | 8 | 42 |
| IgD-λ accompanied with λ | 1 | 0 |

IgA-κ, IgA kappa; IgA-λ, IgA lambda; IgM-κ, IgM kappa; IgM-λ, IgM lambda; IgG-κ, IgG kappa; IgG-λ, IgG lambda; IgD-κ, IgD kappa; IgD-λ, IgD lambda; k, k-free light chain; λ, λ-free light chain.

Supplemental Table 2. Types and distribution of renal pathology in studied groups

|  | MGRS Lesions(n=116) |  | MGUS Lesions(n=231) |
| --- | --- | --- | --- |
| AL amyloidosis | 86 | Membranous nephropathy | 86 |
| Cast nephropathy | 14 | IgA nephropathy | 23 |
| Light chain deposition disease | 9 | Diabetic nephropathy | 20 |
| Type 1 Cryo GN | 2 | Tubulointerstitial nephritis | 19 |
| Light chain proximal tubulopathy | 2 | MPGN | 14 |
| AH amyloidosis | 1 | Minimal change disease | 14 |
| AHL amyloidoses | 1 | Atypical membranous nephropathy | 12 |
| Thrombotic microangiopathy | 1 | Thrombotic microangiopathy | 12 |
|  |  | Focal global glomerulosclerosis | 8 |
|  |  | Lupus nephritis | 8 |
|  |  | Crescent glomerulonephritis | 6 |
|  |  | Ischemic kidney injury | 5 |
|  |  | ANCA-associated vasculitis | 3 |
|  |  | Henoch-Schönlein purpura nephritis | 1 |

Cryo GN, cryoglobulinemic glomerulonephritis; AL amyloidosis, light chain amyloidosis; AH amyloidosis, heavy chain amyloidosis; AHL amyloidoses, heavy chain and light chain amyloidosis; MPGN, membranoproliferative glomerulonephritis.
